# Chylous Ascites Following Thoracic Drainage in a Patient With Previously Undiagnosed Cirrhosis

**DOI:** 10.7759/cureus.115954

**Published:** 2026-09-08

**Authors:** Silvia de Andres Quevedo, Partha Chowdhury

**Affiliations:** 1 Acute Medicine, Queen Elizabeth The Queen Mother Hospital, Margate, GBR

**Keywords:** chest drain, chylous ascites, chylous leak, empyema of the chest, liver cirrhosis, lymphatic hypertension, pleural tap, spontaneous ascites, thoracentesis, thoracic drain complications

## Abstract

We report a rare case of acute chylous ascites developing shortly after insertion of a Seldinger chest drain for left-sided empyema in a man in his 60s with previously unrecognised liver cirrhosis. Pleural fluid cultures grew *Dialister pneumosintes* and *Prevotella intermedia*, consistent with an odontogenic source. Within days, the patient developed rapidly accumulating milky ascites with elevated triglycerides, confirming chylous ascites. Imaging demonstrated previously occult liver cirrhosis, suggesting an underlying predisposition to lymphatic hypertension. The temporal association with thoracic intervention suggested iatrogenic lymphatic disruption in a patient predisposed by cirrhosis. Conservative management with dietary fat restriction and supportive care led to complete resolution. This case emphasises the need to recognise chylous ascites as a potential complication of thoracic procedures and to consider underlying lymphatic vulnerability in patients with occult liver disease.

## Introduction

Chylous ascites results from leakage of triglyceride-rich lymph into the peritoneal cavity due to lymphatic obstruction or structural disruption. While the condition is most often linked to malignancy or cirrhosis [1], iatrogenic causes, particularly following thoracic procedures, are rare.

In liver disease, cirrhosis is a recognised but uncommon aetiology which can increase susceptibility to lymphatic leaks as a result of elevated portal pressure and hepatic lymph production, resulting in lymphatic hypertension [2-4]. Due to the vulnerability of the state, a minor disruption to the lymphatic channels can precipitate a chyle leakage as demonstrated in this case [5]. 

Iatrogenic injury to the thoracic duct or its abdominal tributaries is a documented complication of thoracic and upper abdominal medical procedures [4,6]. However, such injuries most commonly manifest as chylothorax [7]. The development of intra-abdominal chylous ascites without chylothorax following a thoracic intervention is rare, typically indicating that the lymphatic disruption occurred at or below the level of the diaphragm [8].

We present an unusual case of rapid-onset chylous ascites developing shortly after chest drain insertion for empyema of odontogenic origin [9,10]. This case highlights the uncommon but recognised interaction between thoracic interventions and underlying lymphatic hypertension, emphasising the need to consider unrecognised cirrhosis as a significant predisposing factor that magnifies the impact of minor procedural insults [6].

## Case presentation

A 62-year-old man presented with a six-week history of progressive shortness of breath, pleuritic chest pain, and unintentional weight loss. His symptoms were associated with reduced oral intake due to pain and dyspnoea. He had not sought prior medical attention during this period.

His medical background was significant for heavy alcohol use, estimated at approximately five bottles of wine per week over the preceding five years, and previously up to 30 pints of beer weekly since early adulthood. He had a 100-pack-year smoking history and continued to smoke 10 cigarettes daily. He was a retired train engineer. There was no known history of chronic liver disease or prior ascites.

Investigations and initial management

On admission, he was tachypnoeic and hypoxic, requiring high-flow oxygen for type 1 respiratory failure (arterial blood gas: pH 7.49, pCO₂ 3.3 kPa, pO₂ 8.2 kPa, HCO_3_ 22.5, and a raised lactate of 3.2 mmol/L). Otherwise, he was haemodynamically stable and afebrile (respiratory rate 18/min, oxygen saturation 87% on room air, blood pressure 101/59 mmHg).

Admission bloods (Table 1) showed systemic inflammation. Electrolytes showed hyponatremia (126 mmol/L). Liver function tests were mildly deranged, consistent with underlying chronic liver disease.

**Table 1:** Blood results of the patient during the first admission, second admission and at the three-month follow-up along with the normal ranges. Liver function tests, inflammatory markers, and haemoglobin trend are shown in the table. * On day 7, the patient received a unit of RBC, accounting for the increase in haemoglobin. The patient received antibiotics and supplemental nutrition throughout admission.

Blood test	Day 1	Day 2	Day 3	Day 5	Day 6	Day 7	Day 8	Day 9	Day 10	Day 11	Day 14 (readmission)	Day 15	Day 18	Day 22	Three-month follow-up	Normal ranges
Bilirubin (umol/L)	21	14	13	12	8	9	12	13	10	9	10	5	9	7	6	0-29
ALT (U/L)	80	86	74	82	67	57	53	48	34	32	33	35	32	32	23	0-70
ALP (U/L)	174	167	160	224	201	190	227	228	194	190	218	315	216	266	215	30-130
Albumin (g/L)	14	13	11	12	11	12	14	14	13	14	16	17	24	21	31	35-50
CRP (mg/L)	254	216	208	155	93	58	43	47	44	37	19	20	11	30	9	0-10
White Cell Count (10^9/L)	17.3	10.8	9.3	14.2	22.5	16.8	21.5	14.5	11.5	10.3	15.1	8.5	7.8	6	8.5	4-11
Haemoglobin (g/L)	117	113	95	102	84	77*	90	88	83	90	101	103	107	99	158	130-180

Initial chest X-ray (Figure 1) and CT thorax (Figures 2, 3) showed a cavitating consolidation in the left upper lobe, collapse-consolidation of the left lower lobe, and a loculated air-fluid collection in the left lower zone, suspicious for a loculated hydropneumothorax or lung abscess. Prominent lymph nodes and a background of mild emphysema were also noted. No ascites was identified on initial imaging.

**Figure 1:**
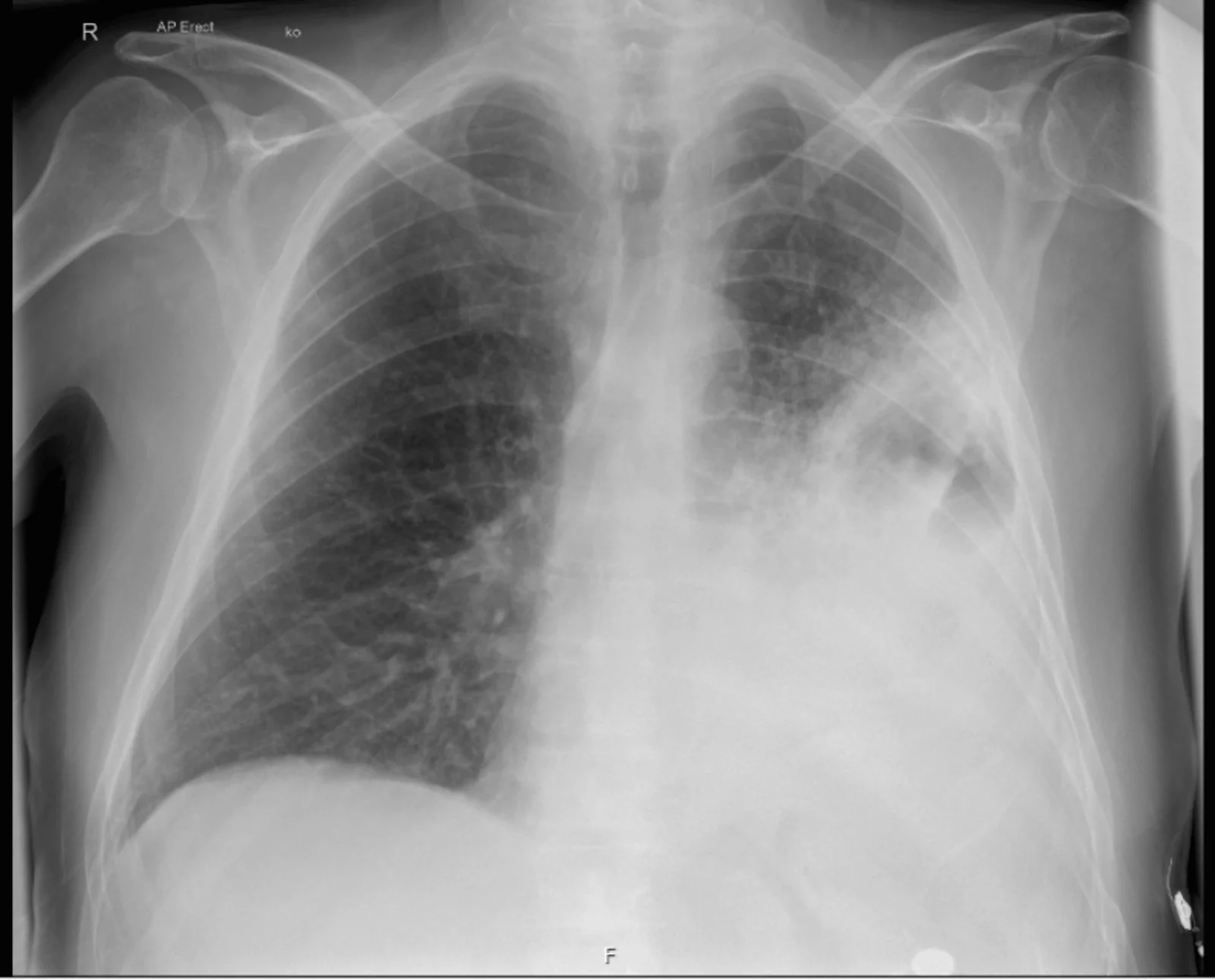
Admission chest X-ray. Left-sided large lung opacity.

**Figure 2:**
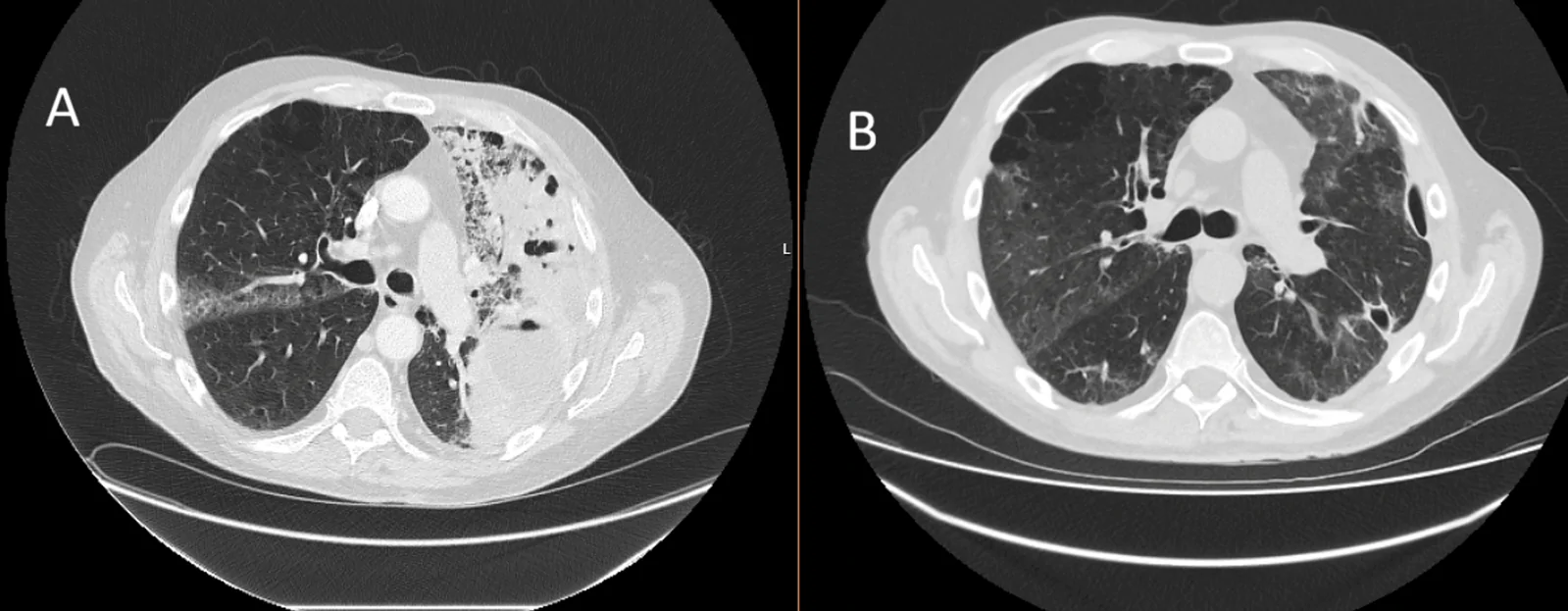
CT thorax on admission (A) and three-month follow-up (B). Admission CT thorax, axial view (A). Report: Left lung ill-defined alveolar opacification in the upper lobe with small cavitating pockets; collapse-consolidation of the lower lobe. Loculated air-fluid collection in the left lower zone consistent with a loculated hydropneumothorax. Right lung: Patchy peripheral ground-glass opacities. Mild bilateral emphysematous changes. Prominent mediastinal lymph nodes; no significant axillary lymphadenopathy. Three-month follow-up CT thorax, axial view (B):  Decreased size of the residual gas and fluid pleural collection on the left. Patchy ground-glass changes within both lungs have also decreased in size. Overall improvement shown on imaging.

**Figure 3:**
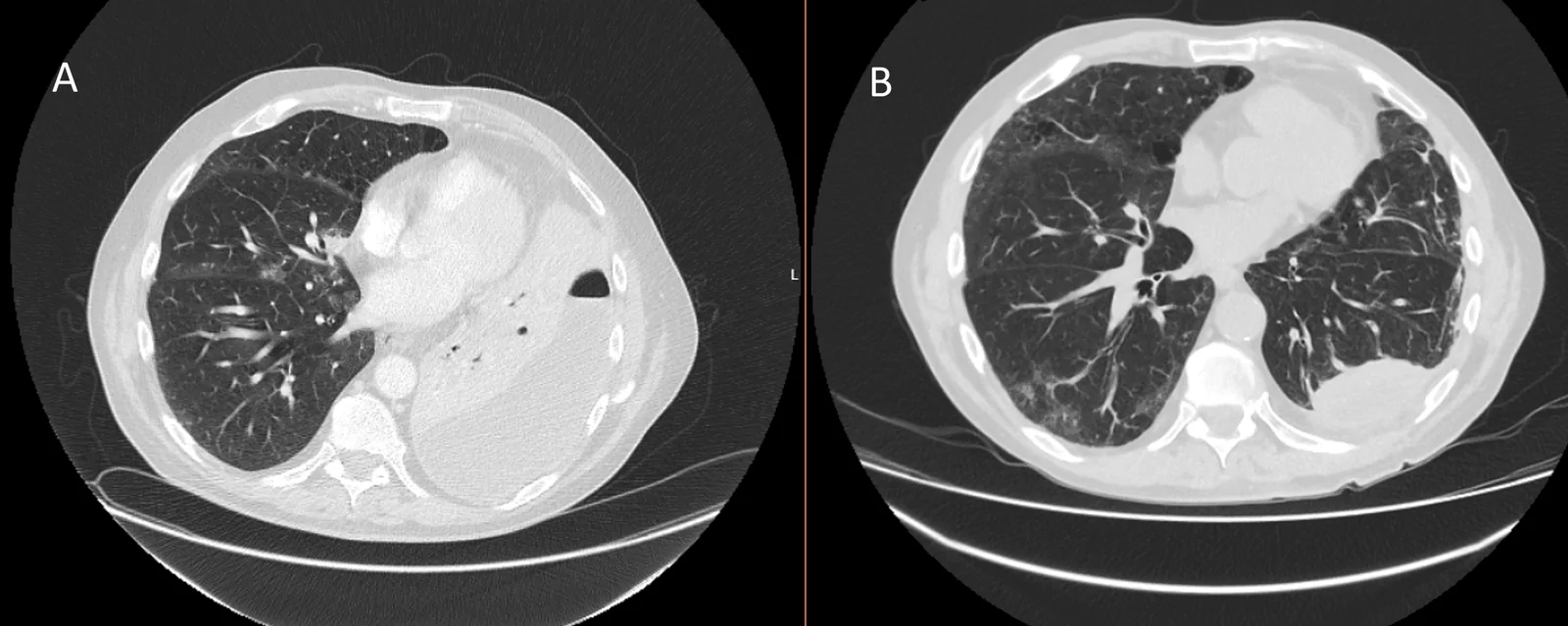
CT thorax on admission (A) and three-month follow-up (B). Admission CT thorax, axial view (A). Radiology report: Left lung showed an ill-defined alveolar opacification in the upper lobe with small cavitating pockets; collapse-consolidation of the lower lobe. Loculated air-fluid collection in the left lower zone consistent with a loculated hydropneumothorax. Right lung: Patchy peripheral ground-glass opacities. Mild bilateral emphysematous changes. Prominent mediastinal lymph nodes; no significant axillary lymphadenopathy. Three-month follow-up CT thorax, axial view (B):  Decrease in the size of the residual gas and fluid pleural collection on the left. The left posterior fluid component has decreased in size. Patchy ground-glass changes within both lungs have also decreased in size.

On day 2 of admission, a left-sided 12F Seldinger chest drain was inserted (Figure 4), and intravenous antibiotic therapy was initiated. 

**Figure 4:**
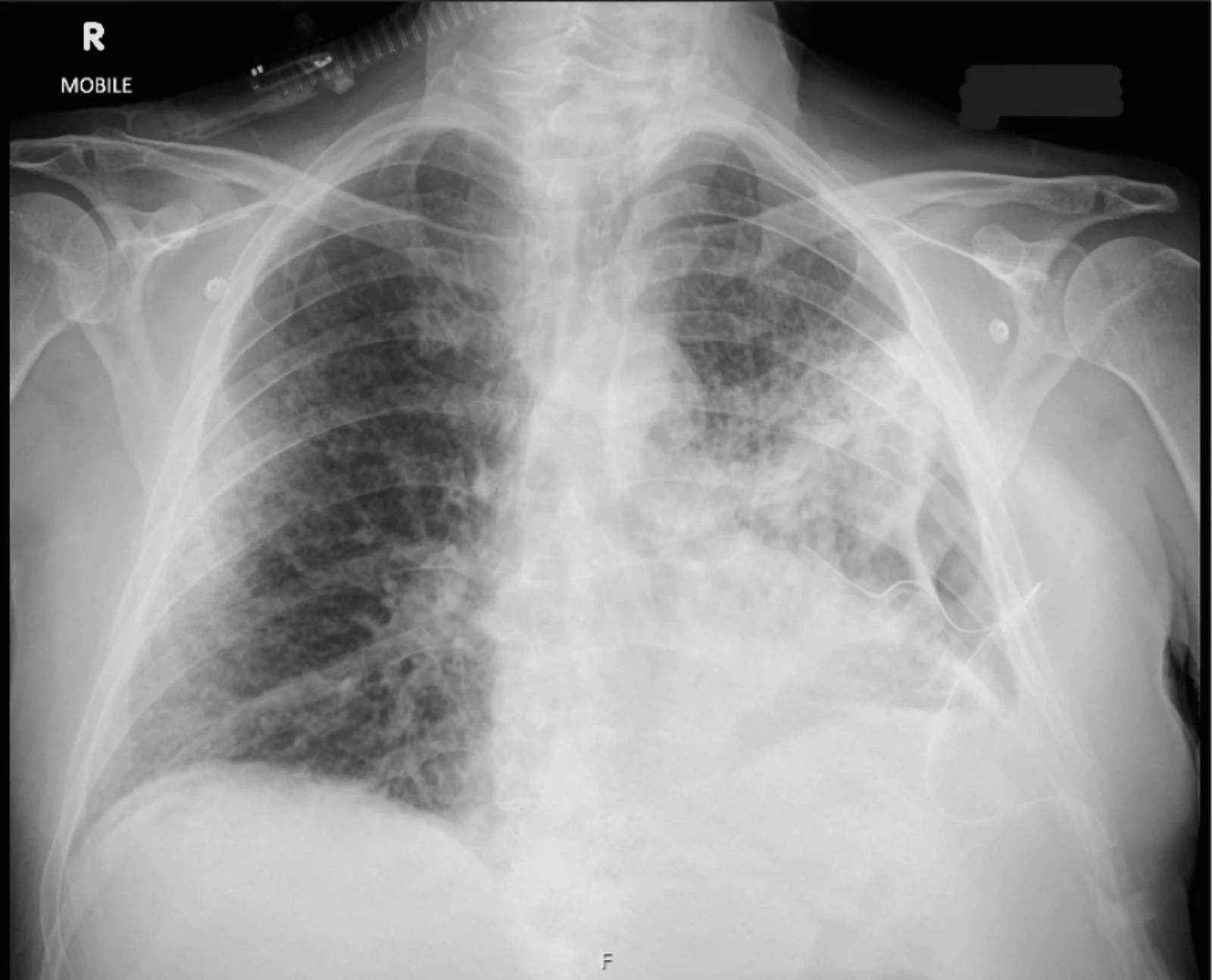
Chest X-ray, immediately following chest drain insertion. Left-sided empyema. Seldinger chest drain visualised on the left lung; tip seen between the 6th and 7th left rib.

In the 24 hours following drain insertion, the patient clinically deteriorated and became haemodynamically unstable, requiring more oxygen and intravenous fluid resuscitation.

Unexpectedly, within three days of chest drain insertion, he developed new-onset abdominal pain, which, alongside deranged liver function tests (day 5 on Table 1), made ruling out a liver abscess essential. Repeat CT imaging demonstrated extensive ascites, which had not been present on prior imaging (Figure 5). This rapid interval development was noted on radiological comparison. Additionally, liver cirrhosis was reported for the first time.

**Figure 5:**
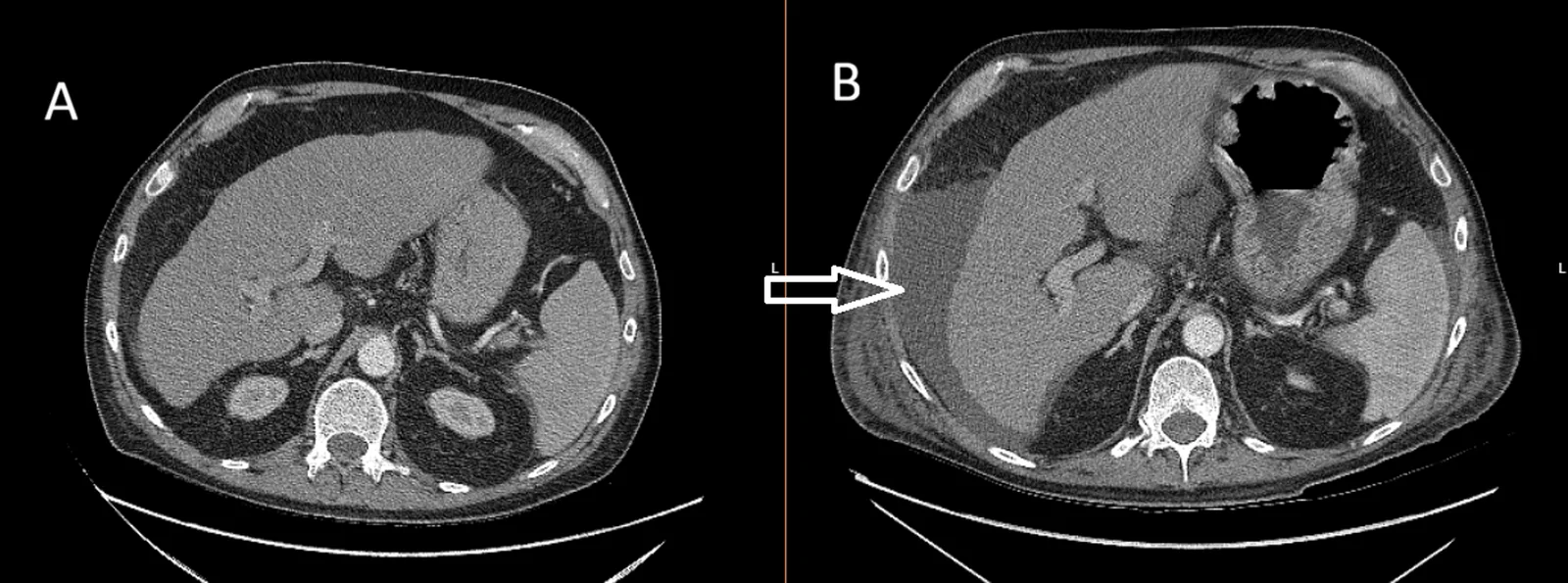
CT abdomen on admission (A) and three days post chest drain insertion (B). Arrow pointing to new ascitic fluid. CT abdomen axial view on admission (A). The visualised upper abdominal structures in the given phase of contrast appear grossly unremarkable. CT abdomen axial view three days post chest drain insertion (B). Radiologist report: Extensive ascites has developed - arrow pointing to the new ascites (confirmed chylous fluid on diagnostic paracentesis tap). There is irregularity of the outline of the liver consistent with cirrhosis. Spleen remains unchanged.

His deterioration continued as he developed hypervolemic hyponatremia, peripheral oedema, and further hypoalbuminemia over the following days. This was considered multifactorial due to the severe infection and the new ascites.

Pleural fluid microbiology identified *Dialister pneumosintes* and *Prevotella intermedia*, and a positive urinary pneumococcal antigen. Given the anaerobic profile, the patient who was initially treated with IV Co-amoxiclav was transitioned to high-dose intravenous amoxicillin and oral metronidazole. It is not commonly described in the literature for *D. pneumosintes* and *P. intermedia* to cause lung empyema, as they are oral flora but can commonly be the culprits of periodontal disease [9]. Due to the likely odontogenic origin of the empyema, either through aspiration (most likely) or haematogenous spread, a mandibular X-ray was performed (Figure 6), which highlighted the link between the growth of these anaerobic bacteria and poor oral health, especially in smokers. 

**Figure 6:**
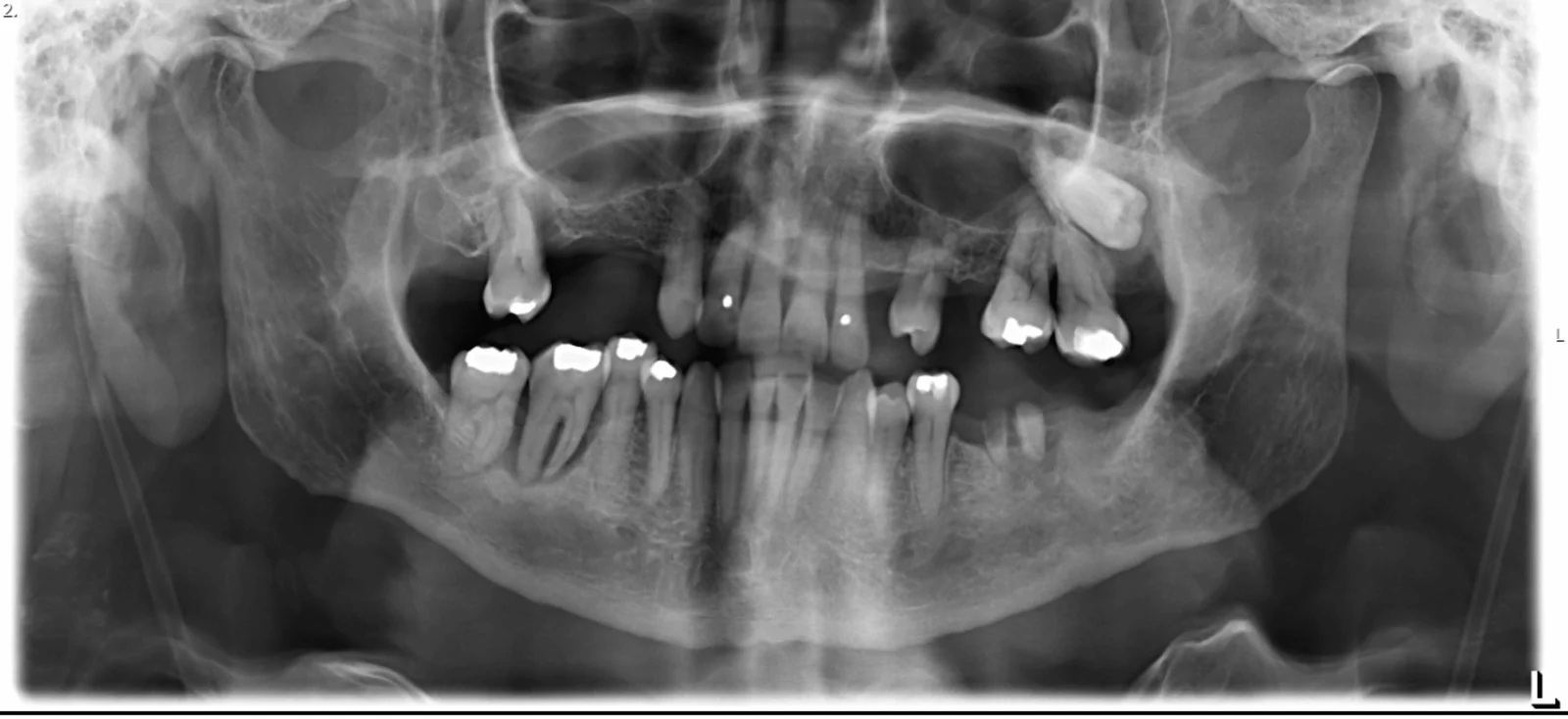
Mandibular X-ray. Radiograph showing evidence of advanced generalised periodontal disease with significant alveolar bone loss, extensive missing dentition, and multiple heavily restored remaining teeth. Findings correlate with a history of poor oral health.

He began to show clinical and biochemical improvement with decreased inflammatory markers (Table 1), and the chest drain, seen laterally displaced on the day 4 chest X-ray (Figure 7), was removed after it stopped swinging on day 5. He was discharged home on day 13.

**Figure 7:**
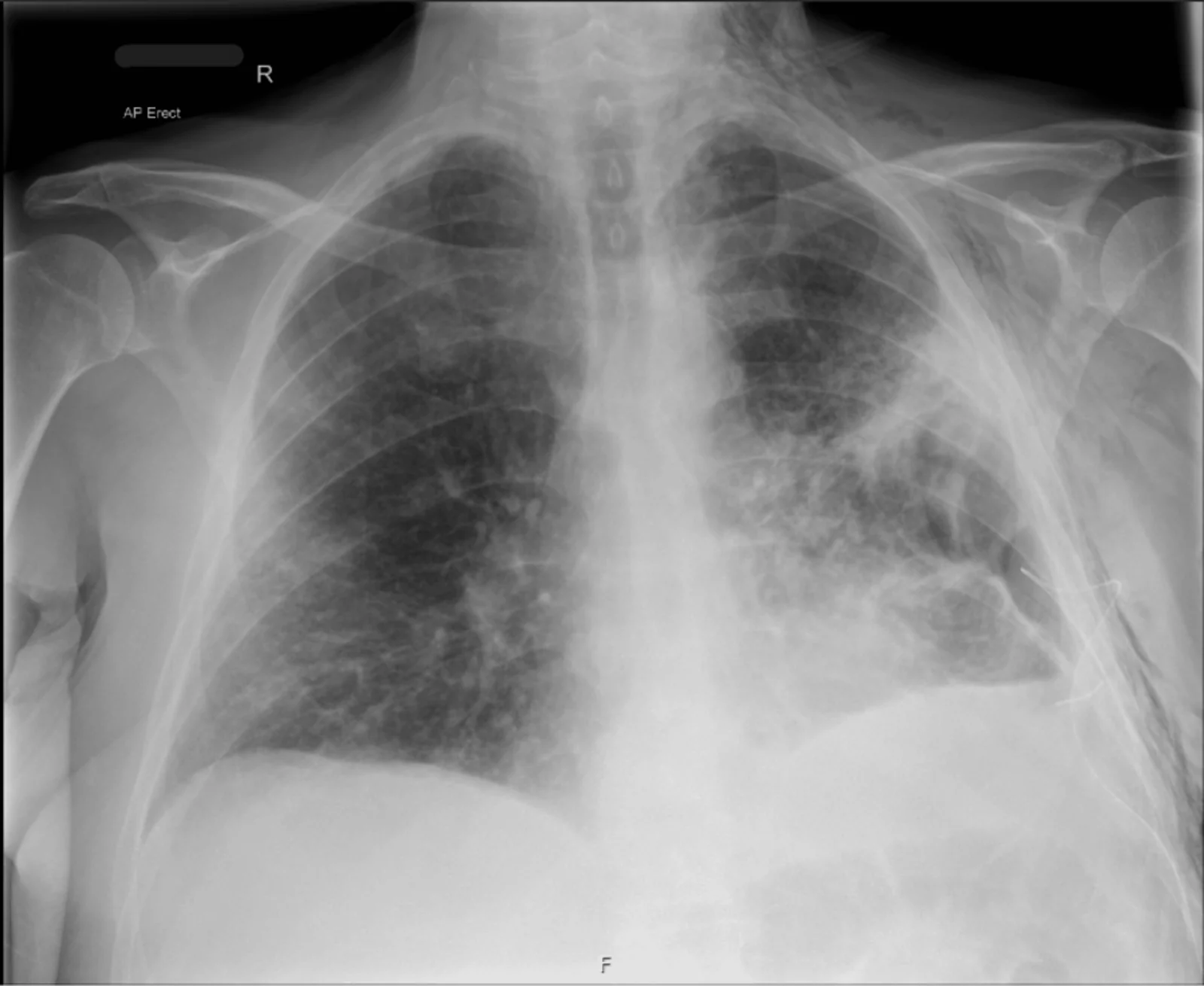
Chest X-ray four days post drain insertion. Tip of drain seen in the lower left lobe. Drain in situ (left) Left-sided empyema decreased in size compared to Figure 4. Seldinger chest drain visualised on the left lung; tip seen displaced laterally, seen between ribs 6 and 7.

Development of chylous ascites

Hours after discharge, he was readmitted with acute abdominal distension and unable to stand. On re-admission, he had progressive abdominal distension, examination revealing large-volume, initially non-tense ascites, which progressed to tense ascites.

Blood results on readmission showed a slight rise in white cells from the discharge bloods (Table 1).

Diagnostic paracentesis on day 4 of readmission (day 17 total) yielded milky fluid. Ascitic fluid triglyceride level was elevated at 2.58 mmol/L, consistent with chylous ascites (threshold >2.4 mmol/L) [11]. SAAG: >11 (serum albumin 24 g/L, ascitic albumin <5 g/L), suggesting a transudative process. Microbiology cultures were sterile.

There was no radiological evidence of intra-abdominal malignancy, bowel perforation, or lymphadenopathy on subsequent imaging. MRI of the liver demonstrated cirrhotic morphology with moderate ascites and mild splenomegaly, without focal hepatic lesions or portal vein thrombosis.

Management of chylous ascites

Repeat CT imaging showed a stable chronic left-sided empyema with interval improvement in pulmonary consolidation. No intra-abdominal source of chyle leak was identified. The ascites persisted post-removal of the drain and continued to leak out of the paracentesis site but remained clinically stable.

Following multidisciplinary discussion with a tertiary thoracic centre, the leading hypothesis was lymphatic system disruption, possibly related to prior left-sided chest drain insertion. He was managed conservatively with gradual reduction of drainage of chylous fluid, dietary fat restriction (to minimise chylomicron production and reduce lymphatic flow through the thoracic duct) [2,5], and antibiotic therapy with consideration for embolisation or surgical ligation if a persistent leak was identified. 

Extensive liver aetiology screening, including viral hepatitis, autoimmune markers, electrophoresis, LDH, and amylase, was negative, with normal bilirubin and improving liver enzymes. MRI liver subsequently confirmed cirrhotic morphology without focal hepatic lesions, with patent portal and hepatic veins, mild splenomegaly, and moderate ascites, as mentioned in the previous section.

However, from the initial presentation, he had no known history of chronic liver disease, with no clinical stigmata of such, no overt portal hypertension, and no Budd-Chiari features on imaging.

Once the drainage became negligible and there was clinical improvement, the drain bag around the paracentesis open site was removed, and there was no further discharge.

Outcome and follow-up

The patient was discharged with a six-week course of oral antibiotics and a low-fat diet. At a three-month follow-up, the patient reported significant functional improvement, with no redevelopment of abdominal distention, resolved breathlessness, and his weight was returning to normal.

Follow-up CT thorax demonstrated a significant decrease in the size of the residual left-sided pleural fluid collection (now measuring 6.4cm x 3.4 cm, previously 15.6cm x 7.3 cm) (Figure 8) and improvement in underlying atelectatic and inflammatory changes. Abdominal imaging showed stable findings of chronic liver disease but complete resolution of the ascitic collection (Figure 9).

**Figure 8:**
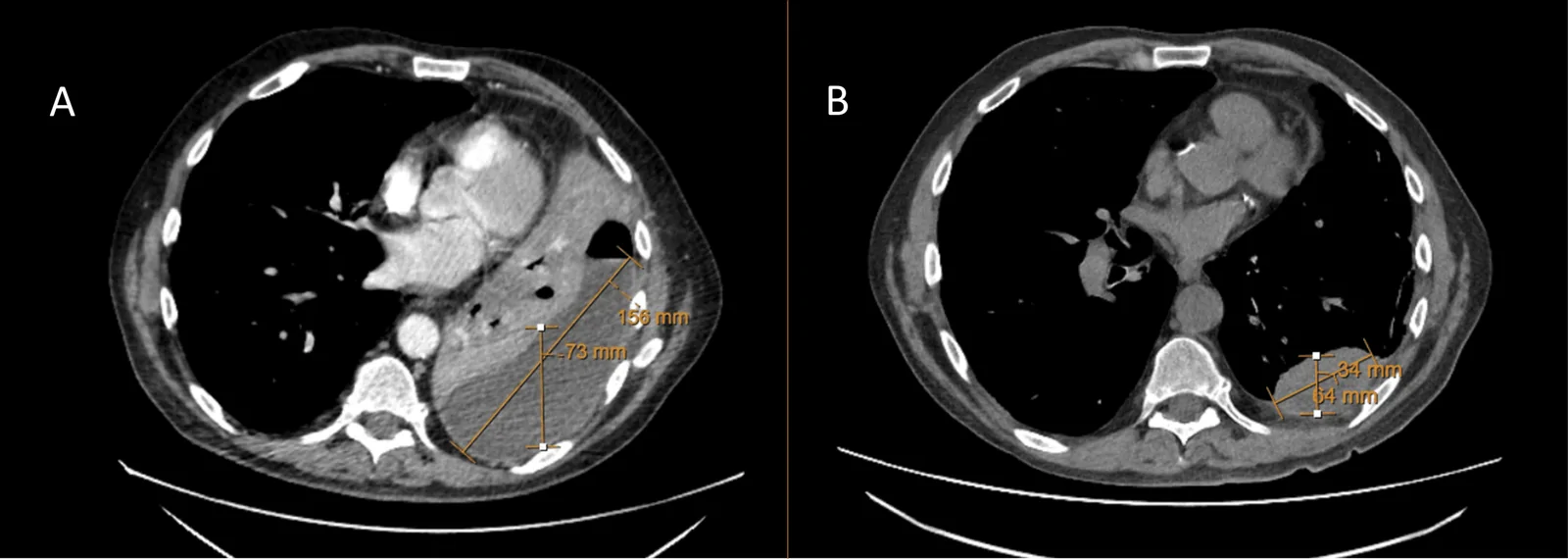
Initial left-sided pleural fluid collection (A). Left-sided pleural fluid collection at the three-month follow-up (B). A: Axial plane of CT thorax on admission. Measurements of left-sided pleural effusion: 73 mm vertically and 156 mm transverse oblique. B: Axial plane of CT thorax on a three-month follow-up. Measurements of left-sided pleural effusion: 34 mm vertically and 64 mm transverse oblique.

**Figure 9:**
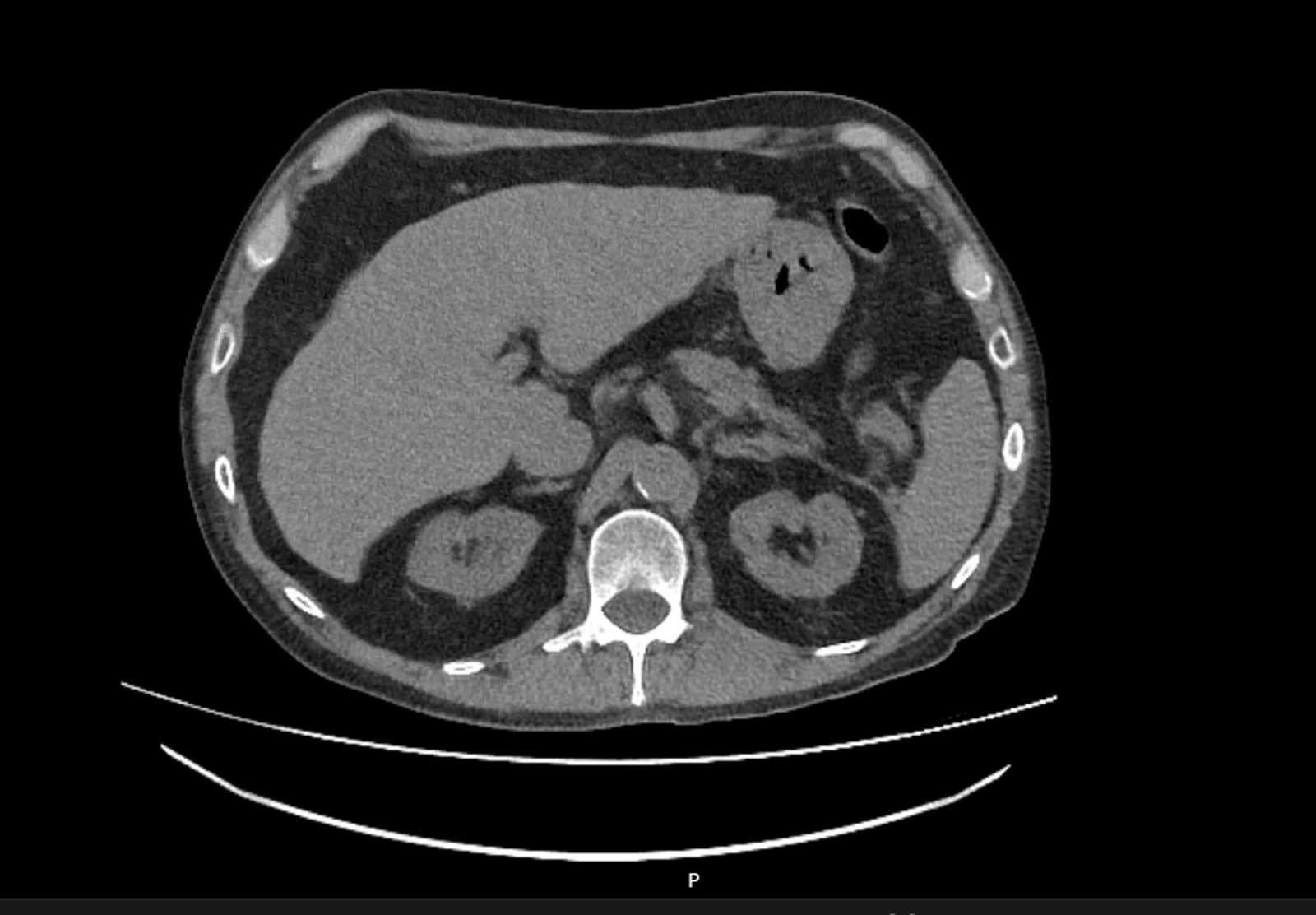
CT thorax at the three-month follow-up showing resolution of ascites

At the outpatient follow-up, the patient was clinically well, with reassuring imaging findings (Figures 2b, 3b, 8b, 9). He was discharged from respiratory follow-up with smoking cessation advice.

## Discussion

This case describes the acute development of chylous ascites shortly after thoracic intervention in a patient with previously unrecognised cirrhosis. Although definitive confirmation of lymphatic injury was not feasible in this case, the temporal relationship between chest drain insertion and the rapid onset of ascites, in the absence of prior intra-abdominal fluid, raises the possibility of an iatrogenic lymphatic leak occurring in a physiologically vulnerable system. Further imaging with lymphangiography may have provided additional diagnostic clarity but was not pursued given clinical improvement.

The thoracic duct originates from the cisterna chyli at the L1-L2 level and ascends through the aortic hiatus of the diaphragm at the T12 level before entering the thorax. Although the thoracic duct typically ascends on the right side of the thorax before crossing to the left at the mid-thoracic level, significant anatomical variation exists (Figure 10) [12-14]. Injury to the thoracic duct or its abdominal tributaries is a recognised complication of thoracic and upper abdominal procedures [6,15], more commonly resulting in chylothorax. In this case, the absence of chylothorax and the presence of isolated chylous ascites suggest that the site of disruption was at or below the level of the diaphragm [8]. This may reflect injury not to the main thoracic duct itself, but to its abdominal tributaries, diaphragmatic lymphatic channels, or, less likely, the cisterna chyli, leading to chyle accumulation within the peritoneal cavity. 

**Figure 10:**
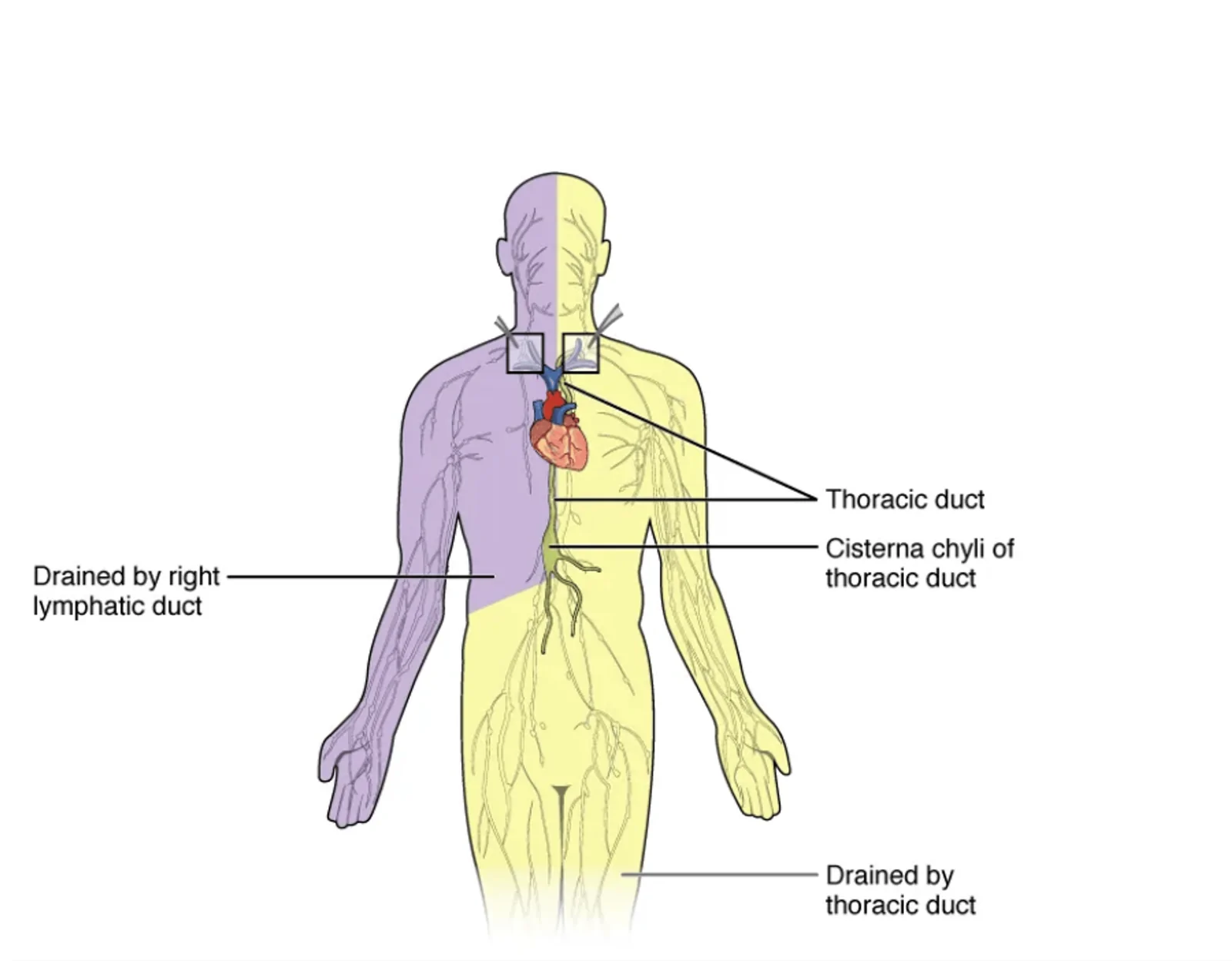
Anatomy of the thoracic duct and trunks Image reproduced under a CC-BY 4.0 license. Source: OpenStax Anatomy and Physiology 2e [12]

While spontaneous chylous ascites can occur in cirrhosis, the acute onset following intervention and subsequent resolution with conservative management favour an iatrogenic lymphatic leak.

Cirrhosis is a well-recognised but uncommon cause of chylous ascites, reported in approximately 0.5% of cases [3]. The underlying mechanism is thought to involve markedly increased hepatic lymph production and elevated portal pressures, resulting in lymphatic hypertension [2,4]. This creates a state in which even minor disruption of lymphatic channels may lead to clinically significant chyle leakage [5]. In this patient, previously undiagnosed cirrhosis likely acted as a predisposing factor, amplifying the effect of any procedural insult.

The rapid onset of large-volume ascites within three days of chest drain insertion, combined with the absence of malignancy, bowel perforation, or alternative intra-abdominal pathology on imaging, supports a multifactorial mechanism. Although iatrogenic injury to the thoracic duct is more commonly associated with chylothorax [7], it is plausible that thoracic intervention altered local pressure dynamics or resulted in minor lymphatic injury, which, in the context of elevated lymphatic flow and low nutritional status, precipitated chylous ascites. The literature has highlighted cases of development of chylous ascites (and pleural effusion) after surgery above the diaphragm (thoracoscopic esophagectomy and gastric tube reconstruction). In such cases, surgery resulted in damage to the thoracic duct, which was then ligated just above the diaphragm. However, the patient developed ascites with lymphangiography evidence of disruption of lymphatic flow at the site of the ligation. Although leakage of the lymphatic drain was not identified, ascites volume exceeded 3000mL in three days, supporting that, although rare, chylous ascites can develop from any damage to the thoracic duct [16]. In our case, while causality cannot be definitively established, the temporal association and clinical course suggest a contributory role of the procedure.

The microbiological findings further provide insight into the underlying pulmonary pathology. The isolation of *Dialister pneumosinte*s and *Prevotella intermedia*, both anaerobic organisms associated with the oral cavity and periodontal disease [9,10], supports an aspiration-related aetiology for the empyema [10,17]. This is consistent with the patient’s significant alcohol use and smoking history, both of which are recognised risk factors for poor oral hygiene and aspiration of oral flora [18]. The identification of these organisms highlights the importance of appropriate anaerobic coverage and targeted microbiological techniques in the management of complicated pleural infections.

## Conclusions

This case highlights the rare potential for chylous ascites to develop following thoracic intervention, particularly in patients with previously unrecognised liver disease. Lymphatic disruption during chest drain insertion may present as isolated intra-abdominal chyle accumulation, while underlying cirrhosis may increase susceptibility to clinically significant lymphatic leakage. Clinicians should therefore consider iatrogenic chylous ascites in patients who develop rapid-onset abdominal distension following thoracic procedures, particularly after common intra-abdominal causes have been excluded.

Diagnosis can be confirmed by measurement of ascitic fluid triglycerides. Conservative management, including dietary fat restriction, may be effective and can allow complete clinical resolution without the need for invasive intervention.
